# The Effect of Weather Variables on Mosquito Activity: A Snapshot of the Main Point of Entry of Cyprus

**DOI:** 10.3390/ijerph17041403

**Published:** 2020-02-21

**Authors:** Katerina Drakou, Thessalia Nikolaou, Marlen Vasquez, Dusan Petric, Antonios Michaelakis, Apostolos Kapranas, Athina Papatheodoulou, Maria Koliou

**Affiliations:** 1 Department of Chemical Engineering, Cyprus University of Technology, 3036 Limassol, Cyprus; katerina.drakou@cut.ac.cy (K.D.); thessalia.nikolaou@hotmail.com (T.N.); athina.papatheodoulou@gmail.com (A.P.); 2 Faculty of Agriculture, Laboratory for Medical and Veterinary Entomology, University of Novi Sad, 21000 Novi Sad, Serbia; dusanp@polj.uns.ac.rs; 3 Department of Entomology & Agricultural Zoology, Benaki Phytopathological Institute, 14561 Athens, Greece; a.michaelakis@bpi.gr (A.M.); a.kapranas@bpi.gr (A.K.); 4 Ministry of Health, 1148 Nicosia, Cyprus; mkoliou@spidernet.com.cy

**Keywords:** temperature, relative humidity, precipitation, *Ae. caspius*, *Ae. detritus*, *Cx. pipiens*, surveillance, *Aedes* invasive species

## Abstract

Mosquitoes are vectors of pathogens, causing human and animal diseases. Their ability to adapt and expand worldwide increases spread of mosquito-borne diseases. Climate changes contribute in enhancing these “epidemic conditions”. Understanding the effect of weather variables on mosquito seasonality and host searching activity contributes towards risk control of the mosquito-borne disease outbreaks. To enable early detection of *Aedes* invasive species we developed a surveillance network for both invasive and native mosquitoes at the main point of entry for the first time in Cyprus. Mosquito sampling was carried out for one year (May 2017–June 2018), at bimonthly intervals around Limassol port. Morphological and molecular identification confirmed the presence of 5 species in the study region: *Culex. pipiens*, *Aedes detritus*, *Ae. caspius*, *Culiseta longiareolata* and *Cs. annulata.* No invasive *Aedes* mosquito species were detected. The Pearson’s correlation and multiple linear regression were used to compare number of sampled mosquitoes and weather variables for three most numerous species (*Cx*. *pipiens*, *Ae. detritus* and *Ae. caspius*). The population densities of the most numerous species were highest from February to April. Number of *Cx. pipiens* (−0.48), *Ae. detritus* (−0.40) and *Ae. caspius* (−0.38) specimens sampled was negatively correlated with average daily temperature. Monthly relative humidity showed positive correlation with the numbers of the species sampled, *Cx. pipiens* (0.66) *Ae. detritus* (0.68), and *Ae. caspius* (0.71). Mosquito abundance of *Cx. pipiens* (0.97) *and Ae. detritus* (0.98) was strongly correlated to seasonal precipitation as well. Our work is a stepping stone to further stimulate implementation of International Health Regulations and implementation of early warning surveillance system for detection of invasive *Aedes* mosquitoes, native mosquitoes and arboviruses they may transmit. A network for the surveillance of both invasive and native mosquito species at the main point of entry for the first time in Cyprus was developed. Number of mosquitoes sampled was correlated with weather factors to identify parameters that might predict mosquito activity and species distribution to the prevention of international spread of vector mosquitoes and vector-borne diseases.

## 1. Introduction

In recent decades, international travel and trade raise globally and this may lead to increased transportation of potential insect vectors to new countries and greater risk of transmission of both human and animal diseases. Mosquitoes are vectors of pathogens causing malaria, filariasis, West Nile and Zika diseases, Dengue and Chikungunya fever [1,2]. Mosquitoes of *Culex* genera are important vectors of arboviruses and filarial nematodes, creating severe public health problems worldwide during the last decades [3]. Detrimental vector-borne diseases (VBD) specific to the invasive *Aedes* mosquitoes are an increasing burden worldwide. *Aedes albopictus*, also known as the Asian tiger mosquito, is a highly invasive species, classified as one of the world’s top hundred worst invasive species [4]. It has a vector capacity for a wide range of vector-borne diseases such as Chikungunya, Dengue, Yellow fever and Zika [5,6,7]. It was first introduced in Europe through international transport routes for used tires and ‘lucky bamboo’ [8] and has been reported in 31 European countries [9].

Recent reported cases regarding mosquito-borne viruses like Chikungunya, Dengue and West Nile virus cause great concern to public health authorities in Europe [10,11]. Surveillance programs are required to early detect invasive species, monitor and assess mosquito population dynamics of native ones. Autochthonous neuroinvasive human West Nile virus infection was reported for the first time in Cyprus in 2016 [12]. In 2019, the total recorded cases for WNV increased to 23, with two deaths [13]. The presence and establishment of invasive mosquito species have not been confirmed on the island to date [14].

Recent studies show that the spread, activity and longevity of vector mosquitoes might be affected by climatic changes [15,16]. The main weather factors that influence mosquito seasonal activity, possibly by altering their developmental, reproductive and mortality rates are the temperature, relative humidity and precipitation [17,18,19]. Temperature and relative humidity have been shown to affect mosquito activity positively, however, the effect depends on the mosquito species and the microclimate [20,21,22]. High precipitation affects the quality and quantity of mosquito breeding habitats causing either increase to their population by maintaining their breeding sites or decrease, by flushing out mosquito larvae from their breeding sites [21,23]. Unrevealing the relationship between weather factors and mosquito activity can provide valuable information in order to tailor surveillance and control programs.

Cyprus, the third biggest island in the Mediterranean Sea and well-known tourist destination, is being affected by the political conditions in neighbouring countries increasing immigrants, such as asylum-seekers and refugees in the last decade. Based on quarterly asylum report, provided by the European Commission, Cyprus records the highest rate of registered first-time applicants during the first quarter of 2019 [24]. At the same time, Cyprus has an increase of 20% in the number of tourists during the last decade [25].

Cyprus has 23 native mosquito species, belonging to five genera (*Anopheles*, *Aedes*, *Culex*, *Culiseta*, *Uranotaenia*), all identified via morphological identification methods [26]. Nowadays, it is widely accepted that morphological identification is time-consuming and requires high taxonomic expertise. Furthermore, species-level identifications are prone to bias due to misidentifications resulting from the fact that some specimens are damaged, “dirty” or have similar morphological characters [27,28]. DNA barcoding techniques can overcome these limitations and can be applied as a complementary identification method [29].

To prevent international spread of vector mosquitoes and VBD, International Health Regulations advise to the states parties to develop and maintain core capacities in invasive mosquito surveillance at designated international airports and ports. We decided to approach this important international assignment timely.

In this study, a network for the surveillance of both invasive and native mosquito species at the main point of entry was established for the first time. Number of mosquitoes sampled was correlated with weather factors to identify significant parameters to predict mosquito activity and species distribution.

## 2. Materials and Methods

### 2.1. Surveillance Network and Mosquito Identification

**Figure 1:**
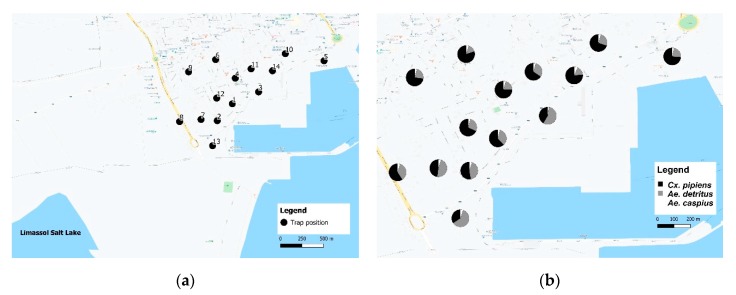
Mosquito trapping sites (**a**) and species composition at each site (**b**).

Adult mosquitoes were collected using CO_2_-baited surveillance traps (BG-Sentinel ©, Biogents, Germany with BG-lure ^®^ and 1 kg dry ice/day) which attract mainly host-seeking females. Traps were placed around the port of Limassol (Figure 1), within grids of 300 m^2^, at ground level according to the manufacturer’s instructions. The location of the traps was based on potential mosquito microhabitat (e.g., irrigation pipes, orchard with figs, olive trees, tree citrus fruits, tires, vegetation). Bimonthly, traps were operating in 24-h cycles and situated in 14 different sites from May 2017 to June 2018 (Table S1). Coordinates of each trapping site were collected using a handheld GPS unit (Montana 680 ©, Garmin, Schaffhausen, Switzerland) and imported into a Geographic Information System software (QGIS Standalone Installer Version 3.4).

Μosquitoes were sampled, transported to the laboratory and stored dry at −20 °C, before analysis. Mosquitoes were morphologically identified using identification keys [30] with a stereoscope (Optika Microscopy, SN 471516, SZN-5, Bergamo, Italy). The damaged and “dirty” ones were randomly chosen for molecular analysis. Composite images (Optika Microscopy, SN 468680, Nikon Eclipse Ci-L, Bergamo, Italy) were taken of representative specimens from each species. The representative specimens were then pinned as voucher specimens and stored at room temperature. Vouchers are available in the Department of Chemical Engineering, Cyprus University of Technology.

### 2.2. Molecular Analysis

From all sampled mosquitoes fifty, the damaged and “dirty” ones, were randomly selected for further investigation using molecular methods. Abdomen and legs were removed from each mosquito. Total DNA was extracted using a commercial kit following manufactures instructions (DNeasy Blood & Tissue kit-QIAGEN, Valencia, CA, USA). A 652-bp fragment of the mitochondrial COI gene was amplified using the following primers: LCO1490 (5′-GGTCAACAAATCATAAAGATATTGG-3′) and HCO2198 (5′-TAAACTTCAGGGTGACCAAAAAATCA-3′) [29]. The designed “universal” primers called LCO1490, and HCO2198 amplified this region was chosen by the Barcode of Life Database (BOLD), as a standard marker [28,31]. Polymerase chain reactions (PCR) were performed in 25 μL of the final volume, containing: Taq DNA Polymerase, dNTPs, MgCl_2_ (2X KAPA LongRange HotStart ReadyMix-KAPA Biosystems, Massachusetts, USA), 0.5 μM of each primer, and ~100 mg DNA template. The thermal cycling steps were as follows: 95 °C initial denaturing for 3 min; 35 cycles of 95 °C of denaturing during 30 s, 48 °C annealing for 30 s, and 72 °C extension for 1 min; and a final extension at 72 °C for 10 min. Samples were then held at 4 °C on the thermocycler until collection. The amplified products and size standards were run on a 1.5% agarose gel stained with a DNA-binding dye (Midori Green-NIPPON Genetics, Germany). The gels were visualised and photographed using a Documentation Camera (INFINITY-1500/36M, Ottawa, Ontario, Canada). PCR products were purified using isopropanol precipitation. The method is as follows: 5 μL of 3 M sodium acetate (NaOAc) pH 5.2 and 50 μL isopropyl alcohol to 50 μL PCR product was added and the mix was incubated at −20 °C for at least 2 h. Following centrifuge (14,000 rpm, 30 min), the precipitate was washed with 70% cold ethanol and centrifuged again (14,000 rpm, 5 min). Then it was dissolved in 30 μL of ddH20. The purified PCR products were sent for cycle-sequencing for both the forward and reverse direction using the same primers (Macrogen, Inc., Amsterdam, Netherlands).

### 2.3. Weather Variables

The meteorological data of temperature, relative humidity and precipitation were obtained from the closest meteorological station at approximately 6 km west of trapping sites, located at Akrotiri Peninsula [32]. Daily and monthly averages were calculated from daily values of T_min_ and T_max_, relative humidity and precipitation (Table 1).

### 2.4. Statistical Analysis

Mosquito abundance was related to meteorological factors (temperature, relative humidity and precipitation), measured for the period 2017–2018. Multiple linear regression analysis was performed to measure the overall effect of all-weather variables on mosquito activity and parameters with a *p*-value less than 0.05 were considered significant to explain the mosquito abundance [33]. Pearson correlation coefficient, r, was used to determine the strength and direction of a linear relationship, between two variables. An r-value between 1.0 to 0.9 was considered very strong, 0.89 to 0.7 strong, 0.69 to 0.4 moderate and 0.39 to 0.1 weak linear correlation [34,35].

## 3. Results

In total, 1917 mosquitoes were collected during the one-year survey (May 2017–June 2018), out of which 1036 were classified as *Cx. pipiens*, 671 as *Ae. detritus*, 77 as *Ae. caspius* and 133 remained unidentified. Apart from these three species, molecular identification revealed the presence of *Culiseta longiareolata* and *Cs. annulata* in 50 specimens, randomly chosen from the pool of 133 unidentified, damaged individuals. No invasive mosquito species were detected.

### 3.1. Influence of Weather on Mosquito Seasonality and Daily Activity

**Figure 2:**
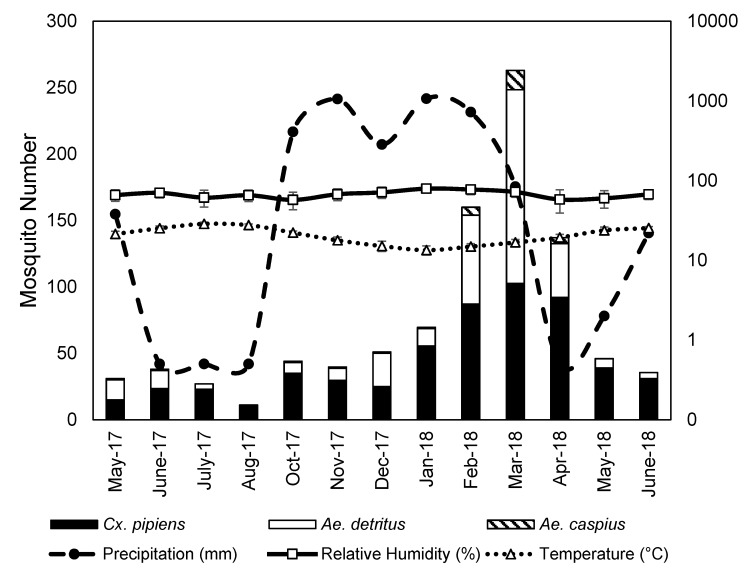
Relationships between weather variables and mosquito seasonal activity. The secondary *Y*-axis illustrates the values of three weather variables (temperature, relative humidity and precipitation).

The total number of mosquito specimens belonging to the three most numerous species was highest during January, February, March and April (Figure 2). When T_max_ of the day exceeded 28 °C, a decrease in the mosquito abundance was observed. Based on Pearson correlation coefficient and multiple linear regression analysis, our results showed best fit to daily averages of temperature, and monthly values of humidity and precipitation. The average temperature in the day of capture showed a moderate negative correlation with *Cx. pipiens* (−0.48) and *Ae. detritus* (−0.40), and weak with *Ae. caspius* (−0.38). Monthly average relative humidity showed a moderate positive correlation with number of sampled *Cx. pipiens* (0.66) and *Ae. detritus* (0.68), and a strong positive correlation with *Ae. caspius* (0.71). A significant very strong positive correlation was recorded between precipitation values and number of sampled *Cx. pipiens* (0.97) and *Ae. detritus* (0.98) specimens. Results were not significant for the number of sampled *Ae. caspius* (0.94) specimens (Table 1), possibly due to low numbers present in the study area. The highest number of mosquitoes of each species was recorded in March, when the average temperature was 17 °C (T_max_ = 21 °C, T_min_ = 13 °C), the relative humidity 72% and the precipitation 84mm (Figure 2).

### 3.2. DNA Barcode Analysis

Morphological based methods classified undamaged mosquitoes into three species. DNA barcoding was applied to 50 randomly selected specimens’ DNA sequences, were compared with published data in BOLD. The results revealed the presence of five species, *Cx. pipiens* (54%), *Ae. detritus* (35%), *Ae. caspius* (4%), *Culiseta longiareolata* (6%) and *Culiseta annulata* (1%). All sequences showed above 97.5% similarity with the COI gene when compared with databases on BOLD. Hence the species identification was considered trustworthy.

## 4. Discussion

The aim of our research was to implement International Health Regulations and early warning surveillance system for detection of invasive *Aedes* mosquitoes timely. Also, the system will serve for surveillance of native mosquitoes and arboviruses they transmit (e.g., WNV). To do that, we developed a network for the surveillance of both invasive and native mosquito species at the main point of entry for the first time in Cyprus. Number of mosquitoes sampled was correlated with weather factors to identify parameters that might predict mosquito activity and species distribution. We hope that our work will contribute to the prevention of international spread of vector mosquitoes and VBD.

Environmental variables such as temperature, relative humidity and precipitation are known to impact mosquito activity, survival and distribution. Our results demonstrate that temperature significantly affects host searching activity of *Cx. pipiens*, *Ae. detritus* and *Ae. caspius* population. Precisely, temperatures between 15 °C to 24 °C seem to be more suitable for their host searching activity. Higher abundance in that period might be direct consequence of the preceding precipitation providing the multiple breeding sites. Temperatures above 28 °C lead to decrease of the abundance. These results are consistent with previous studies that showed temperatures between 15 °C to 28 °C to be more favourable for mosquitoes and that temperatures greater than 30 °C increase mosquito mortality [21,23,36,37]. It is reported that high temperatures cause intense metabolic rate leading to low respiration rate and finally to death [36,38]. The Pearson coefficient correlation confirms the negative relationship between temperature and mosquito activity. Relative humidity in the study area ranges between 57% to 79%. Statistical analysis shows a significant moderate positive relationship between monthly relative humidity and number of sampled *Cx. pipiens* and *Ae*, *detritus.* Significant strong positive relationship was observed for *Ae. caspius*. It has been reported that high humidity increases egg production, larval indices, mosquito activity and influences their activities [18,19]. Other studies have shown that a suitable range of humidity stimulating mosquito flight activity is between 44% and 69%, with the most appropriate reaching 65% [19,23]. During the year of the survey, the annual precipitation was 284 mm in the study area. Our results show a robust positive significant correlation between precipitation and mosquito activity. These results are consistent with previous studies that demonstrate the substantial impact of precipitation on mosquito population growth [15,19,23,39], through the activation of their breeding sites.

It is well known that environmental variables are interrelated, and for that reason, it is complicated to assess each factor separately. Further, other factors seem to play an important role in mosquito activity and abundance. Ferraguti et al. (2016) show that mosquito density and species composition was affected by anthropogenetic landscape transformation [40]. Möhlmann et al. (2017) notice specific differences in mosquito abundance and diversity in relationship with different geographical latitudes [41].

DNA sequencing and morphology-based identification results show the presence of 5 mosquito species in the study area. *Culex pipiens* and *Ae. detritus* are the most abundant species in the area. 

By tradition, the *Culex pipiens* complex consists of several species, subspecies, forms, races, physiological variants or biotypes according to various authors. At present, it includes the names *Cx. pipiens* Linnaeus, *Cx. pipiens* biotype molestus Forskal, *Cx. quinquefasciatus* Say, *Cx. pallens* Coquillett, *Cx. restuans* Theobald and *Cx. torrentium* Martini (considered as a separate species because of the genetical distance to *Cx. pipiens*) in the Holarctic as well as two Australian members, *Cx. australicus* Dobrotworsky and Drummond and *Cx. globocoxitus* Dobrotworsky [42].

The status of the three first names has been taxonomically established by the designation of neotypes [43,44,45]. It is now generally accepted that the former *Cx. pipiens* biotype molestus [44] is not separated from *Cx. pipiens* (former *Cx. pipiens* biotype pipiens) and is designated as a biotype as no individually diagnostic genetic differences have been found [46,47]. However, European populations of both *Cx. pipiens* and *Cx. pipiens* biotype molestus are phylogenetically separated [48,49], which is congruent to the finding that it is not possible to induce autogeny by supernutrition of biotype pipiens larvae [50].

The females of the complex are complicated to separate the field material. In several reared populations, it took eight variables and discriminant analysis to discern between pipiens, molestus and quinquefasciatus females, and overlapping was considerable [51].

*Culex quinquefasciatus* Say and *Cx. pallens* Coquillet have been considered as subspecies of *Cx. pipiens* [52]. They freely hybridise in areas of overlapping distribution but show a difference in the male hypopygial morphology [51]. *Culex pipiens* and *Cx. torrentium* are two separate sibling species [43,52] defined by genetic characteristics [53,54] and different morphology in some life stages.

In his latest review, Harbach (2012) suggested the use of pipiens Assemblage when referring to the taxon traditionally known as the *Culex pipiens* complex, to avoid difficulties associated with the meaning of the word “complex” [55]. He concluded that *Cx. pipiens* and *Cx. quinquefasciatus* are separate species; *Cx. molestus* is an ecological and physiological variant of *Cx. pipiens*; and *Cx. pallens*, being a *Cx. pipiens* and *Cx. quinquefasciatus* hybrid in introgression areas has no taxonomic status under the provisions of the International Code of Zoological Nomenclature. Based on morphological similarity, the Harbach Pipiens Assemblage includes *Cx. pipiens*, *Cx. quinquefasciatus* and perhaps *Cx. australicus.*

Among the species identified, *Cx pipiens* has the most significant medical significance due to its capability to transmit WNV [56]. It is known that *Cx. pipiens* is an endemic vector of Southern Europe for West Nile virus. Therefore, these findings are of great importance, pointing out the necessity to take strict measures in order to control or suppress its population and subsequently prevent the spread of mosquito-borne diseases. *Aedes detritus* and *Ae. caspius* are of lesser medical importance but are well-known major molestants in many European countries [3,14,26]. As an aid to safeguard the early detection of invasive mosquito vectors, surveillance programs should be continuing in the island, especially in the main entry points, the ports, the international airports, as well as the regions in which the Republic of Cyprus does not exercise effective control. Furthermore, virus surveillance programs should be conducted for the early detection of pathogens transmitted by native species (e.g., WNV) and reporting to public health services to increase mosquito control awareness.

## 5. Conclusions

Morphology and molecular identification showed the presence of 5 mosquito species in the study area: *Cx. pipiens*, *Ae. detritus*, *Ae. caspius*, *Culiseta longiareolata* and *Culiseta annulata.* The results of this study showed that the populations of *Cx. pipiens*, *Ae. detritus* and *Ae. caspius* was highest from February to April, as a consequence of precipitation yearly distribution. Correlation between number of sampled *Cx. pipiens*, *Ae. detritus* and *Ae. caspius* females and average daily temperatures was negative, and positive when related to monthly average relative humidity and precipitation. The seasonality results indicate that in most of the Mediterranean countries which have climate similar to Cyprus surveillance should not be restricted to April–October period, but should encompass extended the whole year.

## Figures and Tables

**Table 1:** Pearson coefficient correlation (r-values) and Multiple linear regression analysis (*p*-values) values between numbers of mosquito sampled and daily average temperature, monthly average relative humidity, and monthly precipitation (May 2017–June 2018).

Environmental Variables	*Cx. Pipiens*		*Ae. Detritus*		*Ae. Caspius*	
	r-Value	*p*-Value	r-Value	*p*-Value	r-Value	*p*-Value
Daily Average Temperature (°C)	−0.4896	0.0030 *	−0.4046	0.0240 *	−0.3803	0.0387 *
Monthly Average Relative Humidity (%)	0.6658	0.0356 *	0.6849	0.0289 *	0.7118	0.0209 *
Monthly Precipitation (mm)	0.9746	0.0254 *	0.9831	0.0169 *	0.9446	0.0554

R = 1.0−0.9 (Very Strong Correlation), r = 0.89−0.7 (Strong Correlation), r = 0.69−0.4 (Moderate Correlation), r = 0.39−0.1 (Weak Correlation), *p* < 0.05 (significant) *.

## Supplementary Materials

The following are available online at https://www.mdpi.com/1660-4601/17/4/1403/s1, Table S1: The total number of mosquitoes collected from each trap (May 2017 to June 2018), Table S2: Weather data (May 2017 to June 2018).
