# Supplementary material for: The Effect of Weather Variables on Mosquito Activity: A Snapshot of the Main Point of Entry of Cyprus
Source: Int J Environ Res Public Health. 2020 Feb 21;17(4):1403. doi: 10.3390/ijerph17041403 (PMC7068582; doi:10.3390/ijerph17041403)
Supplement: Supplementary file 1 [file ijerph-17-01403-s001.pdf]

International Journal of
Environmental Research and Public Health

MDPI

Article

The effect of weather variables on mosquito activity: A snapshot of the main point of entry of Cyprus

Katerina Drakou [1], Thessalia Nikolaou [1], Marlen Vasquez [1,*], Dusan Petric [2], Antonios Michaelakis [3], Apostolos Kapranas [3], Athina Papatheodoulou [1] and Maria Koliou [4]

[1] Department of Chemical Engineering, Cyprus University of Technology, Limassol, Cyprus; katerina.drakou@cut.ac.cy (K.D.); ta.nikolaou@edu.cut.ac.cy (T.N.); athina.papatheodoulou@gmail.com (A.P.)
[2] Faculty of Agriculture. Laboratory for Medical and Veterinary Entomology, University of Novi Sad, Novi Sad, Serbia; dusanp@polj.uns.ac.rs (D.P.)
[3] Department of Entomology & Agricultural Zoology, Benaki Phytopathological Institute, Athens, Greece; a.michaelakis@bpi.gr (A.M.); a.kapranas@bpi.gr (A.K.)
[4] Ministry of Health, Nicosia, Cyprus; mkoliou@spidernet.com.cy (M.K.)
* Correspondence: marlen.vasquez@cut.ac.cy

Received: date; Accepted: date; Published: date

Supplementary Materials: The following are available online at www.mdpi.com/xxx/s1.

Table S1. The total number of mosquitoes collected from each trap (May 2017 to June 2018).

Traps	GPS Coordinates		Species			
	Latitude	Longitude	*Cx. pipiens*	*Ae. detritus*	*Ae. caspius*	Unidentified
1	33.00667	34.65085	105	58	7	13
2	33.00478	34.64871	112	89	11	18
3	33.00998	34.65235	80	109	4	7
4	33.00705	34.65408	105	29	6	10
5	33.01823	34.65628	42	13	2	5
6	33.00457	34.65644	16	3	1	2
7	33.00273	34.64887	59	58	6	10
8	33.00003	34.64860	65	40	5	9
9	33.00116	34.65489	86	27	5	8
10	33.01335	34.65718	63	23	4	6
11	33.00904	34.65527	109	50	7	12
12	33.00471	34.65157	97	39	6	12
13	33.00419	34.64555	73	128	11	18
14	33.01173	34.65502	23	5	2	4

Table S2. Weather data (May 2017 to June 2018).

Date	Relative Humidity (%)	Max Temp (C)	Min Temp (C)	Precipitation (mm)
5/1/2017	83	24.7	17.3	0.0
5/2/2017	76	23.0	16.0	0.0
5/3/2017	64	23.4	14.8	0.0
5/4/2017	66	23.3	17.2	0.0
5/5/2017	76	23.3	15.2	0.0
5/6/2017	77	24.0	16.5	0.0
5/7/2017	79	23.9	17.7	0.0
5/8/2017	82	24.7	17.3	0.0
5/9/2017	54	28.7	18.2	0.0
5/10/2017	41	31.2	18.1	0.0
5/11/2017	72	24.1	18.7	0.0

5/12/2017	70	26.0	16.4	0.0
5/13/2017	39	28.4	17.7	0.0
5/14/2017	63	28.9	19.1	0.0
5/15/2017	71	29.2	19.3	0.0
5/16/2017	61	29.0	18.0	0.0
5/17/2017	76	29.5	19.5	0.0
5/18/2017	68	28.2	21.1	0.8
5/19/2017	48	24.6	18.4	2.8
5/20/2017	71	22.7	18.2	0.0
5/21/2017	72	23.8	17.2	0.0
5/22/2017	74	23.8	16.1	0.2
5/23/2017	60	23.5	18.2	0.0
5/24/2017	61	24.6	14.5	0.0
5/25/2017	72	24.3	16.1	0.0
5/26/2017	72	24.7	13.7	0.0
5/27/2017	52	28.2	15.8	0.0
5/28/2017	63	26.0	16.7	0.0
5/29/2017	69	25.2	16.1	0.0
5/30/2017	50	25.4	17.1	0.0
5/31/2017	63	24.3	17.1	0.0
6/1/2017	57	26.6	17.0	0.0
6/2/2017	45	30.1	20.5	0.0
6/3/2017	83	29.7	19.6	0.0
6/4/2017	68	30.7	20.1	0.0
6/5/2017	60	27.7	19.0	0.0
6/6/2017	65	27.1	18.1	0.0
6/7/2017	71	29.6	19.1	0.0
6/8/2017	71	31.2	20.9	0.0
6/9/2017	74	30.2	22.1	0.0
6/10/2017	80	27.4	22.1	0.0
6/11/2017	80	28.1	19.8	0.0
6/12/2017	82	28.6	20.5	0.0
6/13/2017	72	26.4	19.5	0.0
6/14/2017	74	27.3	20.9	0.0
6/15/2017	70	28.9	20.6	0.0
6/16/2017	70	31.0	20.4	0.0
6/17/2017	59	30.1	21.4	0.0
6/18/2017	78	28.4	22.4	0.0
6/19/2017	77	27.7	22.0	0.0
6/20/2017	74	27.3	21.6	0.0
6/21/2017	62	27.4	19.3	0.0
6/22/2017	56	28.4	19.7	0.0
6/23/2017	68	30.9	20.7	0.0
6/24/2017	65	32.5	21.7	0.0
6/25/2017	73	35.0	22.5	0.0
6/26/2017	68	33.4	23.5	0.0
6/27/2017	58	32.6	23.2	0.0
6/28/2017	74	30.1	23.2	0.0
6/29/2017	82	31.0	23.7	0.0
6/30/2017	91	33.3	23.1	0.0
7/1/2017	26	37.4	25.2	0.0
7/2/2017	48	38.5	25.4	0.0
7/3/2017	54	36.0	26.0	0.0
7/4/2017	49	31.7	24.4	0.0
7/5/2017	64	32.5	22.5	0.0
7/6/2017	73	30.5	21.2	0.0

7/7/2017	76	32.6	23.3	0.0
7/8/2017	55	33.6	22.8	0.0
7/9/2017	32	35.2	24.1	0.0
7/10/2017	34	33.8	23.3	0.0
7/11/2017	54	34.4	23.7	0.0
7/12/2017	44	35.4	24.3	0.0
7/13/2017	72	34.4	25.9	0.0
7/14/2017	79	32.1	23.8	0.0
7/15/2017	80	32.3	24.4	0.0
7/16/2017	79	31.6	24.6	0.0
7/17/2017	71	32.0	22.1	0.0
7/18/2017	65	32.8	24.0	0.0
7/19/2017	59	30.6	25.9	0.0
7/20/2017	68	30.7	23.1	0.0
7/21/2017	77	31.0	22.2	0.0
7/22/2017	74	30.8	24.0	0.0
7/23/2017	65	32.7	24.4	0.0
7/24/2017	74	33.2	25.9	0.0
7/25/2017	55	34.3	25.5	0.0
7/26/2017	37	34.1	25.1	0.0
7/27/2017	63	34.0	25.5	0.0
7/28/2017	68	30.9	24.6	0.0
7/29/2017	66	31.6	24.2	0.0
7/30/2017	71	33.2	24.1	0.0
7/31/2017	66	35.1	23.4	0.0
8/1/2017	55	35.8	24.5	0.0
8/2/2017	78	31.7	24.6	0.0
8/3/2017	75	31.1	24.4	0.0
8/4/2017	76	30.8	25.3	0.0
8/5/2017	66	31.4	22.4	0.0
8/6/2017	72	31.9	24.2	0.0
8/7/2017	75	31.6	23.9	0.0
8/8/2017	59	32.5	23.8	0.0
8/9/2017	61	31.9	23.8	0.0
8/10/2017	54	31.2	22.9	0.0
8/11/2017	74	31.2	25.0	0.0
8/12/2017	76	31.8	24.7	0.0
8/13/2017	72	31.4	24.1	0.0
8/14/2017	77	32.1	23.8	0.0
8/15/2017	69	31.8	23.6	0.0
8/16/2017	55	31.5	21.8	0.0
8/17/2017	69	31.6	21.5	0.0
8/18/2017	66	31.4	22.8	0.0
8/19/2017	71	31.0	23.6	0.0
8/20/2017	67	31.0	22.8	0.0
8/21/2017	64	31.3	23.2	0.0
8/22/2017	73	31.2	24.3	0.0
8/23/2017	63	32.5	23.5	0.0
8/24/2017	69	32.8	24.4	0.0
8/25/2017	57	33.2	24.7	0.0
8/26/2017	31	35.8	24.3	0.0
8/27/2017	34	32.8	22.8	0.0
8/28/2017	70	30.5	23.3	0.0
8/29/2017	69	30.3	22.5	0.0
8/30/2017	63	30.2	20.8	0.0
8/31/2017	72	30.6	22.5	0.0

9/1/2017	66	31.2	23	0
9/2/2017	56	32.8	22.7	0
9/3/2017	69	33	23.1	0
9/4/2017	47	34.4	23.7	0
9/5/2017	45	31.9	22.8	0
9/6/2017	70	30.1	23.4	0
9/7/2017	55	31.6	21.2	0
9/8/2017	57	32.3	20.6	0
9/9/2017	56	32.5	20.6	0
9/10/2017	63	31	21.7	0
9/11/2017	58	31.8	21.2	**0**
9/12/2017	71	32.5	22.6	**0**
9/13/2017	65	32	21.1	0
9/14/2017	70	31.3	22	0
9/15/2017	76	31.4	22.9	0
9/16/2017	74	30.8	24.3	0
9/17/2017	78	29.6	23	0
9/18/2017	77	30	23.7	0
9/19/2017	54	30	21.3	0
9/20/2017	57	29.1	22.3	0
9/21/2017	76	31.1	22.4	0
9/22/2017	55	27.9	24.4	0
9/23/2017	60	29.1	20.1	**0**
9/24/2017	62	29.2	20	**0**
9/25/2017	64	28.6	20.7	0
9/26/2017	70	27.8	21.9	0
9/27/2017	63	27.7	19.9	0
9/28/2017	62	28.3	19.7	0
9/29/2017	65	28.8	19.7	0
9/30/2017	67	28.6	22.1	0
10/1/2017	68	27.2	19.8	0.0
10/2/2017	55	29.0	19.8	0.0
10/3/2017	60	28.1	20.4	0.0
10/4/2017	60	28.7	20.3	0.0
10/5/2017	57	28.0	18.6	0.0
10/6/2017	42	28.2	17.5	0.0
10/7/2017	57	28.1	18.4	0.0
10/8/2017	62	27.5	18.7	7.0
10/9/2017	65	24.4	20.0	0.0
10/10/2017	76	25.5	17.9	0.0
10/11/2017	66	25.6	16.2	0.0
10/12/2017	59	27.0	16.8	0.0
10/13/2017	32	27.9	16.0	0.0
10/14/2017	40	26.5	15.1	0.0
10/15/2017	50	26.8	16.9	0.0
10/16/2017	33	27.8	18.3	0.0
10/17/2017	38	28.7	17.4	0.0
10/18/2017	35	29.3	16.9	0.0
10/19/2017	36	28.3	15.7	0.0
10/20/2017	40	28.7	16.7	0.0
10/21/2017	48	26.4	14.3	0.0
10/22/2017	66	25.4	17.9	0.0
10/23/2017	72	25.8	17.0	0.0
10/24/2017	73	25.6	17.2	3.8
10/25/2017	87	25.8	19.1	22.8
10/26/2017	70	26.9	16.9	0.4

10/27/2017	63	28.0	16.1	0.0
10/28/2017	72	25.7	18.6	4.8
10/29/2017	67	25.2	17.7	2.2
10/30/2017	65	23.6	17.0	0.0
10/31/2017	64	23.6	17.6	0.0
11/1/2017	70	20.1	14.3	0.0
11/2/2017	65	24.0	13.6	0.0
11/3/2017	66	24.2	16.2	0.0
11/4/2017	71	22.3	14.1	40.2
11/5/2017	88	20.9	14.5	2.0
11/6/2017	75	22.8	12.7	0.0
11/7/2017	72	23.1	13.8	0.0
11/8/2017	51	23.6	14.5	0.0
11/9/2017	57	23.4	11.0	0.0
11/10/2017	76	23.3	13.0	0.0
11/11/2017	70	23.4	12.4	0.0
11/12/2017	67	22.8	12.7	0.0
11/13/2017	62	25.5	19.5	0.0
11/14/2017	78	23.5	17.2	0.0
11/15/2017	76	24.4	14.2	0.0
11/16/2017	60	24.1	14.2	0.0
11/17/2017	53	25.2	12.6	0.0
11/18/2017	45	25.5	13.6	0.0
11/19/2017	79	22.4	16.1	4.2
11/20/2017	91	21.4	14.6	29.0
11/21/2017	45	19.3	12.7	0.2
11/22/2017	60	17.9	11.2	0.0
11/23/2017	66	20.4	8.1	0.0
11/24/2017	69	20.6	10.0	0.0
11/25/2017	78	19.5	10.9	0.0
11/26/2017	59	17.5	15.9	0.0
11/27/2017	59	21.7	12.6	7.4
11/28/2017	68	22.1	14.5	22.4
11/29/2017	88	19.3	13.5	0.4
11/30/2017	59	20.4	11.6	0.0
12/1/2017	71	20.7	10.8	0.0
12/2/2017	76	21.6	11.3	0.0
12/3/2017	55	21.4	10.1	0.0
12/4/2017	66	21.0	11.0	0.0
12/5/2017	56	20.4	10.1	0.6
12/6/2017	83	17.7	12.3	0.2
12/7/2017	51	15.8	5.6	0.0
12/8/2017	56	9.3	6.0	0.0
12/9/2017	89	18.4	7.2	0.0
12/10/2017	73	18.7	11.0	0.0
12/11/2017	73	20.0	8.3	0.0
12/12/2017	61	21.5	10.1	0.0
12/13/2017	54	21.4	8.8	0.0
12/14/2017	58	20.3	8.6	0.0
12/15/2017	79	16.9	12.3	0.0
12/16/2017	78	20.0	10.3	0.2
12/17/2017	83	19.9	13.2	0.0
12/18/2017	87	21.5	13.3	0.0
12/19/2017	85	19.9	14.9	0.0
12/20/2017	76	21.1	13.2	0.0
12/21/2017	74	21.2	11.9	0.0

12/22/2017	66	20.1	15.7	0.0
12/23/2017	83	20.7	16.5	2.8
12/24/2017	54	19.0	12.2	0.8
12/25/2017	59	17.7	6.4	0.0
12/26/2017	65	18.1	6.1	0.0
12/27/2017	78	20.7	9.3	0.0
12/28/2017	71	21.0	14.7	0.0
12/29/2017	79	21.1	10.6	0.4
12/30/2017	90	19.4	10.7	11.8
12/31/2017	81	15.9	12.3	11.6
1/1/2018	84	10.9	7.0	0.0
1/2/2018	82	18.4	11.5	2.8
1/3/2018	94	19.3	13.9	11.8
1/4/2018	88	17.1	12.1	18.0
1/5/2018	91	18.3	8.8	0.0
1/6/2018	74	19.6	10.2	0.0
1/7/2018	68	19.6	8.1	0.0
1/8/2018	65	18.9	7.8	0.0
1/9/2018	85	18.1	8.4	0.0
1/10/2018	84	18.3	9.6	0.0
1/11/2018	78	19.8	13.7	0.0
1/12/2018	83	19.1	10.4	0.0
1/13/2018	80	18.4	8.5	3.0
1/14/2018	82	16.9	9.6	0.0
1/15/2018	80	17.8	9.5	0.0
1/16/2018	66	16.6	8.5	5.6
1/17/2018	97	17.1	9.1	2.2
1/18/2018	73	18.3	12.2	9.0
1/19/2018	52	15.3	9.1	0.0
1/20/2018	69	17.1	4.8	0.0
1/21/2018	75	17.7	10.8	0.0
1/22/2018	67	18.9	14.3	14.4
1/23/2018	75	16.8	12.6	2.6
1/24/2018	93	16.6	11.2	9.6
1/25/2018	78	15.2	10.7	16.4
1/26/2018	91	12.8	7.3	11.6
1/27/2018	81	16.6	5.9	0.0
1/28/2018	83	17.2	7.5	0.0
1/29/2018	82	17.3	7.7	0.0
1/30/2018	73	17.3	5.5	0.0
1/31/2018	86	18.3	7.8	0.0
2/1/2018	85	18.1	8.4	0.0
2/2/2018	75	18.5	8.1	0.0
2/3/2018	85	18.5	8.6	0.0
2/4/2018	84	18.1	9.9	0.0
2/5/2018	83	18.6	11.1	0.0
2/6/2018	73	21.0	11.3	0.0
2/7/2018	89	20.6	10.6	0.0
2/8/2018	55	19.4	14.6	0.0
2/9/2018	68	20.1	10.6	0.0
2/10/2018	83	19.2	13.0	0.0
2/11/2018	72	18.8	16.0	0.0
2/12/2018	78	17.2	14.8	0.0
2/13/2018	78	17.1	9.4	1.6
2/14/2018	80	17.4	11.0	3.4
2/15/2018	82	19.1	9.7	0.0

2/16/2018	72	19.4	11.5	37.0
2/17/2018	96	17.2	11.1	9.4
2/18/2018	42	22.3	13.3	0.0
2/19/2018	70	18.4	7.6	0.0
2/20/2018	81	18.0	11.7	0.4
2/21/2018	86	19.4	10.0	1.2
2/22/2018	77	18.3	12.1	0.0
2/23/2018	78	19.5	11.3	0.0
2/24/2018	70	19.5	8.7	2.4
2/25/2018	74	17.6	12.2	11.2
2/26/2018	96	15.8	11.8	5.6
2/27/2018	74	18.5	11.2	0.2
2/28/2018	80	19.4	9.5	0.0
3/1/2018	86	19.8	10.9	0.0
3/2/2018	67	19.2	8.9	0.0
3/3/2018	71	20.1	9.3	0.0
3/4/2018	79	22.4	10.8	0.0
3/5/2018	77	21.9	12.3	0.0
3/6/2018	82	21.7	16.6	0.0
3/7/2018	84	19.6	12.8	0.0
3/8/2018	86	22.5	14.5	0.0
3/9/2018	72	19.7	16.2	0.0
3/10/2018	67	20.4	13.1	0.0
3/11/2018	77	20.3	10.6	0.0
3/12/2018	81	19.5	10.3	0.0
3/13/2018	79	19.1	10.9	0.0
3/14/2018	82	19.8	13.1	0.0
3/15/2018	75	19.0	11.4	0.0
3/16/2018	60	19.6	13.6	0.0
3/17/2018	60	19.9	9.9	0.0
3/18/2018	76	20.8	13.2	0.0
3/19/2018	65	23.7	13.7	0.0
3/20/2018	84	20.1	16.0	0.0
3/21/2018	83	22.6	13.0	0.0
3/22/2018	83	21.4	12.2	0.0
3/23/2018	55	25.7	16.6	0.0
3/24/2018	64	19.7	15.0	0.2
3/25/2018	69	20.6	13.4	0.0
3/26/2018	62	21.7	11.1	0.0
3/27/2018	49	24.2	10.0	0.0
3/28/2018	48	23.2	15.3	3.8
3/29/2018	68	19.9	13.5	4.4
3/30/2018	69	20.2	13.4	0.0
3/31/2018	73	21.1	10.2	0.0
4/1/2018	68	23.1	13.0	0.0
4/2/2018	71	21.6	11.4	0.0
4/3/2018	69	22.4	12.5	0.0
4/4/2018	42	24.3	10.9	0.0
4/5/2018	36	25.7	10.2	0.0
4/6/2018	35	26.1	12.1	0.0
4/7/2018	67	22.8	12.1	0.0
4/8/2018	66	21.6	14.9	0.0
4/9/2018	71	20.7	12.7	0.0
4/10/2018	71	20.8	11.9	0.0
4/11/2018	65	22.2	12.5	0.0
4/12/2018	83	22.4	14.9	0.0

4/13/2018	81	23.9	14.3	0.0
4/14/2018	69	24.1	15.4	0.0
4/15/2018	85	25.6	15.9	0.0
4/16/2018	30	26.4	14.8	0.0
4/17/2018	32	27.4	14.8	0.0
4/18/2018	23	28.3	14.9	0.0
4/19/2018	45	24.9	14.8	0.0
4/20/2018	76	22.7	17.9	0.0
4/21/2018	64	24.1	15.4	0.0
4/22/2018	28	23.3	15.3	0.0
4/23/2018	37	24.8	12.0	0.0
4/24/2018	54	24.7	14.1	0.0
4/25/2018	30	26.1	17.5	0.0
4/26/2018	51	27.1	18.1	0.0
4/27/2018	60	25.1	17.5	0.0
4/28/2018	58	27.0	17.4	0.0
4/29/2018	78	26.9	17.5	0.0
4/30/2018	81	26.4	18.1	0.0
5/1/2018	46	29.5	17.2	0.0
5/2/2018	48	29.1	21.4	0.0
5/3/2018	44	30.0	23.6	0.0
5/4/2018	46	30.5	21.9	0.0
5/5/2018	79	24.8	20.8	0.0
5/6/2018	75	24.8	19.3	0.0
5/7/2018	72	25.7	18.3	0.0
5/8/2018	59	23.9	16.9	0.0
5/9/2018	69	24.7	18.3	0.0
5/10/2018	77	25.7	17.2	0.0
5/11/2018	51	26.5	17.4	0.0
5/12/2018	75	25.2	17.0	0.0
5/13/2018	59	25.0	16.4	0.0
5/14/2018	73	25.4	16.1	0.0
5/15/2018	64	26.2	15.8	0.0
5/16/2018	77	28.5	17.9	0.0
5/17/2018	57	33.1	19.2	0.0
5/18/2018	22	35.4	21.0	0.0
5/19/2018	28	35.6	20.9	0.0
5/20/2018	27	33.7	24.4	0.0
5/21/2018	55	30.0	21.7	0.0
5/22/2018	56	30.9	22.9	0.0
5/23/2018	55	29.1	19.6	0.0
5/24/2018	71	28.9	21.3	0.0
5/25/2018	69	26.4	21.0	0.0
5/26/2018	69	27.0	18.8	0.0
5/27/2018	69	27.7	19.3	0.0
5/28/2018	69	27.3	19.3	0.0
5/29/2018	61	27.3	18.9	0.0
5/30/2018	69	27.3	19.1	0.0
5/31/2018	70	27.3	19.3	0.2
6/1/2018	78	26.6	20.0	0.2
6/2/2018	62	27.4	19.7	0.0
6/3/2018	70	28.5	19.8	0.0
6/4/2018	68	29.0	19.8	0.0
6/5/2018	67	28.0	19.8	0.0
6/6/2018	48	30.5	19.5	0.0
6/7/2018	53	34.6	20.2	0.0

6/8/2018	35	31.6	21.6	0.0
6/9/2018	62	33.2	21.4	0.0
6/10/2018	76	29.5	24.0	0.0
6/11/2018	67	28.6	22.0	0.6
6/12/2018	75	28.0	21.6	0.2
6/13/2018	76	28.9	21.0	0.0
6/14/2018	75	29.2	23.4	0.0
6/15/2018	70	28.2	21.1	1.2
6/16/2018	85	32.5	22.0	0.0
6/17/2018	66	28.5	23.7	0.0
6/18/2018	70	28.3	22.6	0.0
6/19/2018	67	28.6	22.2	0.0
6/20/2018	66	28.8	20.2	0.0
6/21/2018	71	26.1	21.2	0.0
6/22/2018	69	28.9	22.5	0.0
6/23/2018	72	29.3	23.0	0.0
6/24/2018	74	29.0	20.9	0.0
6/25/2018	73	29.9	22.0	0.0
6/26/2018	61	29.9	22.3	0.0
6/27/2018	68	31.0	22.0	0.0
6/28/2018	68	28.7	24.4	0.0
6/29/2018	65	28.8	23.1	0.0
6/30/2018	66	28.9	21.9	0.0

© 2020 by the authors. Licensee MDPI, Basel, Switzerland. This article is an open access article distributed under the terms and conditions of the Creative Commons Attribution (CC BY) license (http://creativecommons.org/licenses/by/4.0/).
